# SOX17 in Gynecologic Tumors: Biology and Diagnostic Applications

**DOI:** 10.3390/cancers18193162

**Published:** 2026-10-01

**Authors:** Wenfang Liu, Maryam Tahir, Anil V. Parwani, Qingqing Ding, Zaibo Li

**Affiliations:** 1 Department of Pathology, The Ohio State University, Columbus, OH 43210, USA; 2 Department of Pathology, The University of Texas MD Anderson Cancer Center, Houston, TX 77030, USA

**Keywords:** SOX17, PAX8, Müllerian lineage, ovarian carcinoma, endometrial carcinoma, immunohistochemistry, biomarker, angiogenesis, Wnt/β-catenin, digital pathology

## Abstract

SOX17 is a developmental transcription factor that regulates endoderm formation, vascular development, germ cell specification, and tissue differentiation. Recent studies have identified SOX17 as a highly informative immunohistochemical marker and lineage-associated regulator in gynecologic pathology. This review summarizes the physiological functions of SOX17, its expression in normal and neoplastic tissues, and its expanding diagnostic and translational applications. SOX17 demonstrates high sensitivity for most ovarian and endometrial epithelial carcinomas while exhibiting substantially greater specificity than PAX8 because it is consistently absent in the vast majority of renal, thyroid, breast, pulmonary, gastrointestinal, and other nongynecologic carcinomas. These characteristics make SOX17 particularly valuable for supporting Müllerian origin in metastatic tumors and cytology specimens. Emerging evidence also supports its utility in diagnostically challenging settings, including mesonephric-like adenocarcinoma, seminomatous germ cell tumors, yolk sac tumors, angiosarcoma, and selected mesothelial proliferations, where awareness of potential diagnostic pitfalls is essential. Beyond its diagnostic role, SOX17 functions in a context-dependent manner, acting either as a tumor suppressor or as a lineage-survival transcription factor through interactions with signaling pathways such as Wnt/β-catenin and the PAX8 transcriptional network. These biological insights underscore its role in lineage maintenance, angiogenesis, and tumor progression and suggest potential therapeutic relevance. Collectively, current evidence establishes SOX17 as a lineage biomarker with both diagnostic and biologic relevance.

## Simple Summary

SOX17 is a protein that normally helps guide the development of certain organs before birth. In adults, pathologists have discovered that SOX17 is present in some female reproductive organ cancers, especially those arising from the ovary and the lining of the uterus. Because SOX17 is easy to detect with a simple staining test on tissue samples, it has become a valuable tool for identifying the origin of a tumor, particularly when it has spread to another part of the body or when only a small tissue sample is available. This review brings together current knowledge about how SOX17 works in normal and cancerous tissue, compares it to another commonly used marker called PAX8, and highlights situations where SOX17 testing can be misleading. We also summarize the antibody tests pathologists use and highlight open questions, including how SOX17 relates to the molecular subtypes now used to classify uterine cancer.

## 1. Introduction

The SRY-related HMG-box (SOX) family comprises approximately 20 transcription factors that share a highly conserved 79-amino-acid high mobility group (HMG) DNA-binding domain, a defining structural feature responsible for sequence-specific recognition of target genes [1,2,3]. Through regulation of lineage-specific gene expression, SOX proteins govern a wide spectrum of developmental and homeostatic processes across diverse tissues. Their biological activities are highly context dependent, as interactions with distinct cofactors and chromatin environments enable individual SOX proteins to activate or repress different transcriptional programs in a cell type- and developmental stage-specific manner [4,5,6,7,8].

Among these factors, SOX17 is classified within the SOXF subgroup together with SOX7 and SOX18 [9]. Members of this subfamily exhibit overlapping functions during embryogenesis, particularly in vascular and endothelial development [10]. Under physiological conditions, SOX17 is indispensable for the differentiation and maintenance of multiple endodermal and endothelial lineages. It contributes to the formation of numerous organs and tissues derived from the endoderm while also directing the development of mesoderm-derived vascular endothelium [11,12]. During cardiovascular morphogenesis, SOX17 orchestrates key events including endocardial specification, arterial identity, and vascular network formation [13,14]. Beyond the cardiovascular system, it regulates the development of the extrahepatic biliary tract and ventral pancreas [15]. SOX17 also plays an essential role in reproductive biology by promoting primordial germ cell specification, a prerequisite for normal gametogenesis and fertility in both sexes [16].

Aberrant SOX17 expression has been implicated in a broad spectrum of pathological conditions, including cancer [17], infertility [18,19], metabolic disorders such as type 2 diabetes [15,20], and tissue regeneration. Its expression pattern varies across malignancies. In tumor-associated endothelial cells, SOX17 is generally suppressed regardless of cancer type, whereas increased expression has been reported in ovarian carcinoma cells [17]. In endometrial carcinoma, however, published findings remain inconsistent, with studies reporting divergent expression profiles [17,21]. Mechanistically, SOX17 frequently functions as a negative regulator of canonical Wnt/β-catenin signaling, thereby influencing cellular proliferation, differentiation, and tumor progression [22,23].

More recently, SOX17 has emerged as a highly sensitive and specific immunohistochemical biomarker for epithelial neoplasms of the ovary and endometrium [24,25,26,27,28]. Owing to its distinct expression profile and diagnostic performance, it has become an increasingly valuable component of immunohistochemical panels used in gynecologic pathology. This review provides a comprehensive overview of the physiological functions of SOX17, summarizes its expression in normal and neoplastic tissues, and discusses its evolving diagnostic significance in gynecologic oncology, with particular emphasis on its application within contemporary immunohistochemical algorithms.

## 2. Biological Functions of SOX17

SOX17 is a highly conserved SRY-related HMG-box transcription factor that serves as a master regulator of cell fate determination, lineage specification, and tissue differentiation across multiple developmental and disease contexts. During embryogenesis, SOX17 is indispensable for the formation of definitive endoderm and the proper segregation of germ layers. Wnt/β-catenin signaling directly regulates SOX17 expression, and loss of *Sox17* disrupts organizer activity, endoderm development, and embryonic patterning [29]. In addition, SOX17 coordinates gut endoderm morphogenesis and epithelial organization during gastrulation, linking lineage specification with tissue architecture [30]. SOX17 is also the key upstream regulator of human primordial germ cell (PGC) specification, initiating the human germline transcriptional program and establishing PGC identity [16]. In addition, SOX17 contributes to the maintenance and reprogramming of cellular identity in adult tissues. Studies of stem cell biology have demonstrated that SOX17 can confer fetal-type properties on adult hematopoietic stem cells and influence stem cell self-renewal and differentiation programs [31]. More broadly, SOX17 has emerged as an important factor in cellular reprogramming, where it modulates transcriptional networks governing pluripotency, differentiation, and lineage stability [32].

SOX17 plays an essential role in female fertility and uterine function. It is highly expressed in the endometrial epithelium during the window of implantation and regulates pathways required for endometrial receptivity, embryo attachment, and vascular remodeling within the uterus [33]. Reduced SOX17 expression has been associated with impaired implantation capacity, highlighting its importance in establishing a receptive maternal environment for pregnancy [33].

SOX17 plays an essential role in cardiovascular development by regulating endothelial differentiation, vessel maturation and arterial specification [13,14,34]. SOX17 is a key genetic and molecular regulator of pulmonary arterial hypertension (PAH), with pathogenic variants now recognized as an important cause of heritable and pediatric PAH, particularly in patients with congenital heart disease (CHD) [35,36,37,38]. Patients carrying *SOX17* variants typically present at a younger age and often exhibit severe PAH, CHD, and additional vascular abnormalities, supporting the concept of a distinct developmental vascular syndrome [37,39,40]. In addition, SOX17 is a critical endothelial transcription factor that regulates vascular development, angiogenesis, and pulmonary vascular homeostasis [35,41]. Experimental studies have demonstrated that SOX17 deficiency leads to endothelial dysfunction, impaired angiogenesis, metabolic dysregulation, pulmonary vascular remodeling, and endothelial-to-mesenchymal transition (EndMT) through activation of TGF-β/Smad2/3 signaling [42,43,44]. Importantly, reduced SOX17 expression has also been observed in PAH patients without *SOX17* mutations, suggesting that SOX17 deficiency may represent a common pathogenic mechanism across multiple forms of PAH [41].

The developmental functions of SOX17 are frequently co-opted in cancer, where its role can be context dependent. In many malignancies, SOX17 acts as a tumor suppressor through inhibition of Wnt signaling and maintenance of differentiated cellular states, and its expression is often downregulated [22,23]. However, in lineage-dependent cancers such as ovarian carcinoma, SOX17 is upregulated and acquires pro-tumorigenic functions [32]. Recent genomic and functional studies have identified SOX17 as a lineage-survival factor in ovarian cancer that cooperates with the Müllerian lineage transcription factor PAX8 [45,46]. SOX17 and PAX8 form a transcriptional complex that occupies shared enhancer regions and regulates genes critical for ovarian cancer cell proliferation, survival, and maintenance of lineage-specific transcriptional programs [45,46]. This SOX17–PAX8 complex drives tumor angiogenesis by suppressing SERPINE1, an anti-angiogenic factor, and promotes the secretion of pro-angiogenic mediators such as VEGFA and CXCL8, leading to increased endothelial cell recruitment and tumor vascularization [46]. Transcriptomic analyses further demonstrate that the combined expression of SOX17, PAX8, and MUC16 effectively distinguishes ovarian serous cystadenocarcinoma from other tumor types, highlighting its diagnostic and lineage-specific significance [47].

In endometrial carcinoma, however, published findings remain inconsistent, with studies reporting divergent expression profiles [17,21]. One study showed SOX17 is frequently downregulated in endometrial cancer and functions as a tumor suppressor by inhibiting Wnt signaling, preventing β-catenin activation, and thereby limiting uncontrolled cell proliferation [21]. These are relative expression levels compared with normal endometrium; by immunohistochemistry, nuclear SOX17 is retained in the large majority of endometrial carcinomas, so downregulation in this sense does not mean loss of staining. Higher SOX17 expression is associated with longer recurrence-free survival in patients with endometrial cancer [48]. Consistent with these findings, the Cancer Genome Atlas (TCGA) has identified *SOX17* as a driver gene in endometrial carcinoma, reporting multiple recurrent tumor-associated missense mutations [49,50].

A clinically important question left open by these driver-gene data is whether *SOX17* behaves differently across the molecular subtypes of endometrial carcinoma now used for risk stratification. The TCGA-derived, WHO-endorsed classification divides endometrial carcinoma into four groups—*POLE*-mutant (ultramutated), mismatch repair-deficient (MMRd), p53-abnormal, and no specific molecular profile (NSMP)—based on *POLE* exonuclease-domain sequencing together with mismatch repair and p53 immunohistochemistry, and this framework now directly informs prognosis and adjuvant treatment decisions. To date, no published study has systematically stratified SOX17 immunohistochemical expression by these four molecular classes, and this represents an important gap given how central molecular subtyping has become to endometrial cancer management. Indirect, histotype-based observations offer only limited insight: SOX17 is retained with high frequency in serous and clear-cell carcinomas, histotypes that are enriched for p53-abnormal tumors, whereas *POLE*-mutant and MMRd tumors are more often high-grade endometrioid carcinomas in which SOX17 expression is likewise generally preserved by immunohistochemistry; however, this expression has not been quantified within a molecularly annotated cohort. The limits of extrapolating from histotype alone are illustrated by endometrial clear cell carcinoma, in which targeted sequencing shows that *POLE* mutations and mismatch repair protein deficiencies are uncommon and *TP53* alterations predominate instead—underscoring that histologic subtype and molecular class do not map onto one another in a simple, predictable way. Because p53-abnormal tumors carry the poorest prognosis and disproportionately drive endometrial cancer mortality, while *POLE*-mutant and MMRd tumors are increasingly considered for immune checkpoint inhibition, determining whether SOX17 expression tracks with a specific molecular subtype—and whether it adds prognostic information within the NSMP group, which remains the most heterogeneous and hardest to risk-stratify—should be a priority for future correlative studies that integrate SOX17 immunohistochemistry with *POLE* sequencing, mismatch repair testing, and p53 immunostaining.

Collectively, current evidence indicates that SOX17 functions as a master regulator of developmental and tissue-specific transcriptional programs, controlling endoderm formation, germ cell specification, reproductive function, cellular reprogramming, and, in selected cancer contexts, lineage maintenance, angiogenesis, and tumor progression. These findings position SOX17 as both a developmental regulator and a context-dependent oncogenic transcription factor, bridging its physiological roles in endoderm and vascular development with its pathological functions in Müllerian carcinogenesis.

## 3. SOX17 Protein Expression in Normal Tissues

SOX17 expression in normal and reactive tissues provides essential context for interpreting its diagnostic use in gynecologic pathology. In normal tissues, SOX17 is physiologically expressed in Müllerian-derived epithelia, including the fallopian tube, endometrium, and endocervical glands, where it shows strong nuclear staining confined to glandular epithelial cells but not stromal elements [24]. Normal endocervical glands exhibit diffuse, strong nuclear SOX17 staining, whereas ectocervical squamous epithelium is consistently negative, reflecting lineage-specific physiologic expression [24].

Beyond the reproductive tract, SOX17 is also expressed in vascular endothelial cells and germ cell precursors of spermatocytes, consistent with its developmental role in endoderm and vascular differentiation [24].

In contrast, SOX17 expression is absent in most nongynecologic epithelia, such as kidney, thyroid, breast, gastrointestinal, and pulmonary tissues, reinforcing its tissue specificity [24]. A recent study evaluating benign and neoplastic mesothelial lesions demonstrated that SOX17 was negative in all normal mesothelium [51]. Importantly, benign mesothelial cells in cytologic fluids were consistently negative, and endothelial staining was preserved, confirming technical adequacy [26].

## 4. Diagnostic Applications of SOX17 Immunohistochemistry

### 4.1. Endometrial and Ovarian Carcinomas

**Figure 1:**
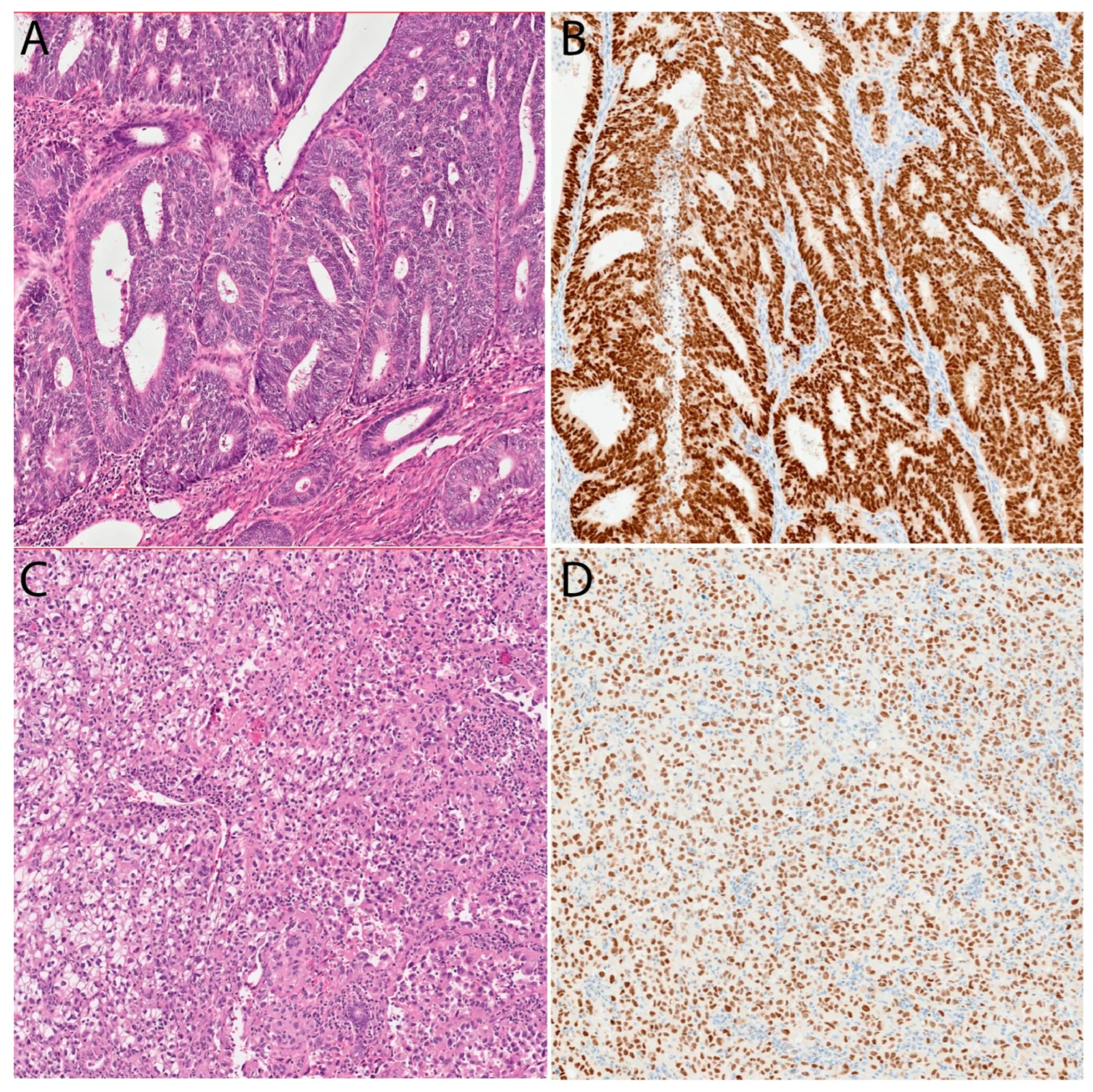
SOX17 expression in endometrial tumors. SOX17 immunostaining showed positive results for nuclear staining in endometrial endometrioid carcinoma (**A**,**B**) and clear cell carcinoma (**C**,**D**). (**A**,**C**) Hematoxylin and eosin staining. (**B**,**D**) SOX17 immunostaining. Original magnification, ×100.

**Figure 2:**
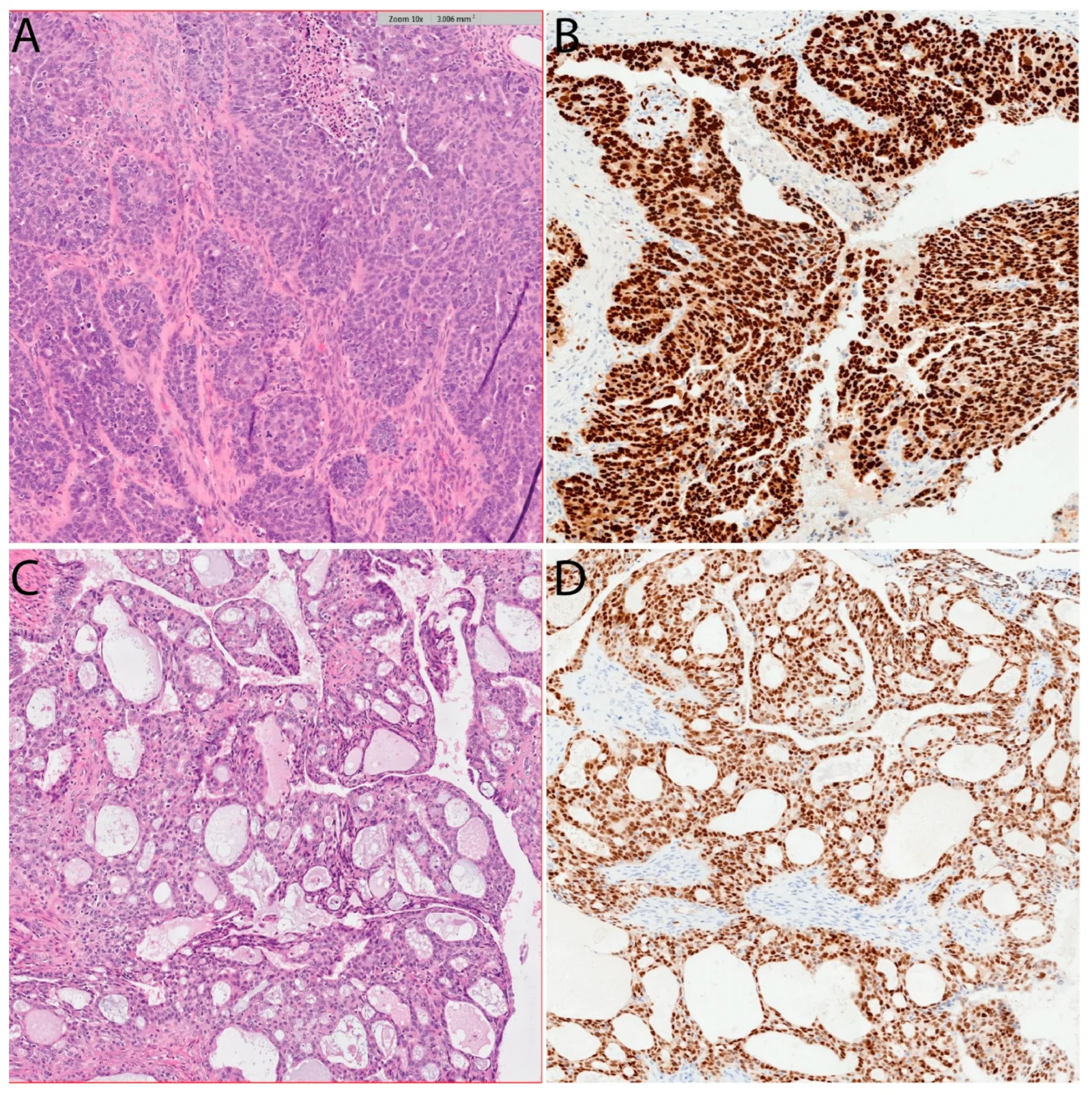
SOX17 expression in ovarian tumors. SOX17 immunostaining showed positive results for nuclear staining in ovarian high-grade serous carcinoma (**A**,**B**) and low-grade serous carcinoma (**C**,**D**). (**A**,**C**) Hematoxylin and eosin staining. (**B**,**D**) SOX17 immunostaining. Original magnification, ×100.

Beyond its biological role in tumor development and differentiation, SOX17 has emerged as one of the most promising diagnostic immunohistochemical markers for identifying tumors of Müllerian origin, particularly ovarian and endometrial epithelial neoplasms [24,25,27,28] (Table 1; Figure 1 and Figure 2). The landmark studies by Shaker et al. and Zhang et al. independently demonstrated that SOX17 is highly expressed in the majority of ovarian serous, endometrioid, and clear cell carcinomas, as well as in endometrial endometrioid, serous, and clear cell carcinomas [24,25]. A major advantage of SOX17 is its superior specificity. Unlike PAX8, which is routinely expressed in renal and thyroid carcinomas and may occasionally be seen in breast carcinoma and others, SOX17 is consistently negative in the overwhelming majority of nongynecologic epithelial tumors [25]. Large studies encompassing more than 1500 nongynecologic tumors demonstrated absent SOX17 expression in breast, lung, colorectal, pancreatic, hepatobiliary, bladder, salivary gland, thyroid, and renal carcinomas, with only rare exceptions [24]. This specificity substantially improves the diagnostic distinction between Müllerian carcinomas and metastatic tumors from kidney or thyroid. Although SOX17 is highly sensitive for most ovarian and endometrial carcinomas, notable subtype-specific differences have been observed. Expression is markedly reduced or absent in ovarian mucinous tumors, with one large series reporting complete absence in all ovarian mucinous carcinomas examined. Similarly, reduced expression has been reported in high-grade neuroendocrine carcinomas and dedifferentiated/undifferentiated endometrial carcinomas [24,27].

The utility of SOX17 extends beyond surgical pathology and has been validated in cytology specimens. Satturwar et al. demonstrated diffuse, strong nuclear SOX17 expression in all 29 metastatic ovarian and endometrial carcinomas examined in effusion and fine-needle aspiration cell blocks, yielding a sensitivity of 100% and a specificity of 98.2% [26]. A subsequent independent study by Shrestha et al. confirmed these findings, reporting SOX17 positivity in all 30 metastatic gynecologic tumors in serous fluid specimens while remaining negative in nearly all nongynecologic tumors [52]. These results support the incorporation of SOX17 into routine cytology workups for metastatic carcinoma of unknown primary, particularly when a gynecologic origin is suspected.

More recent studies have demonstrated that SOX17 is a useful adjunctive immunohistochemical marker for the diagnosis of mesonephric-like adenocarcinoma (MLA), although its expression is variable. Tahir et al. found that most MLAs exhibit loss or decreased expression of SOX17 while diffuse strong PAX8 expression unlike other types of endometrial carcinomas which usually show either strong expression for both SOX17 and PAX8 or strong expression of SOX17/decreased expression of PAX8 [53]. SOX17 with PAX8 significantly improves the identification of endometrial MLA and enhances its distinction from morphologic mimics when interpreted alongside established mesonephric markers such as GATA3, TTF-1, and CD10. Zhang et al. demonstrated similar findings with most MLAs showing absent or low SOX17 expression, although approximately 22% exhibited high expression [54]. The study also showed all mesonephric remnants/hyperplasias were SOX17 negative [54]. More recently, Valencia et al. illustrated SOX17 was negative in an unusual endometrial MLA with prominent clear cell carcinoma-like features, demonstrating that SOX17, together with morphology, a comprehensive immunophenotypic panel, and molecular findings, can help prevent misclassification of MLAs [55]. Overall, current evidence supports SOX17 as a complementary Müllerian biomarker that improves the diagnostic accuracy of MLA when incorporated into a multiparametric immunohistochemical approach [56].

Collectively, the current literature demonstrates that SOX17 is among the more sensitive markers for non-mucinous ovarian and endometrial epithelial carcinomas. Its sensitivity is comparable to that of PAX8, while its specificity is superior because of the lack of expression in renal, thyroid, breast, and most other nongynecologic carcinomas. SOX17 is particularly valuable in metastatic and cytology specimens and is a reasonable addition to immunohistochemical panels used to support Müllerian origin.

### 4.2. Uterine Cervical Tumors

**Figure 3:**
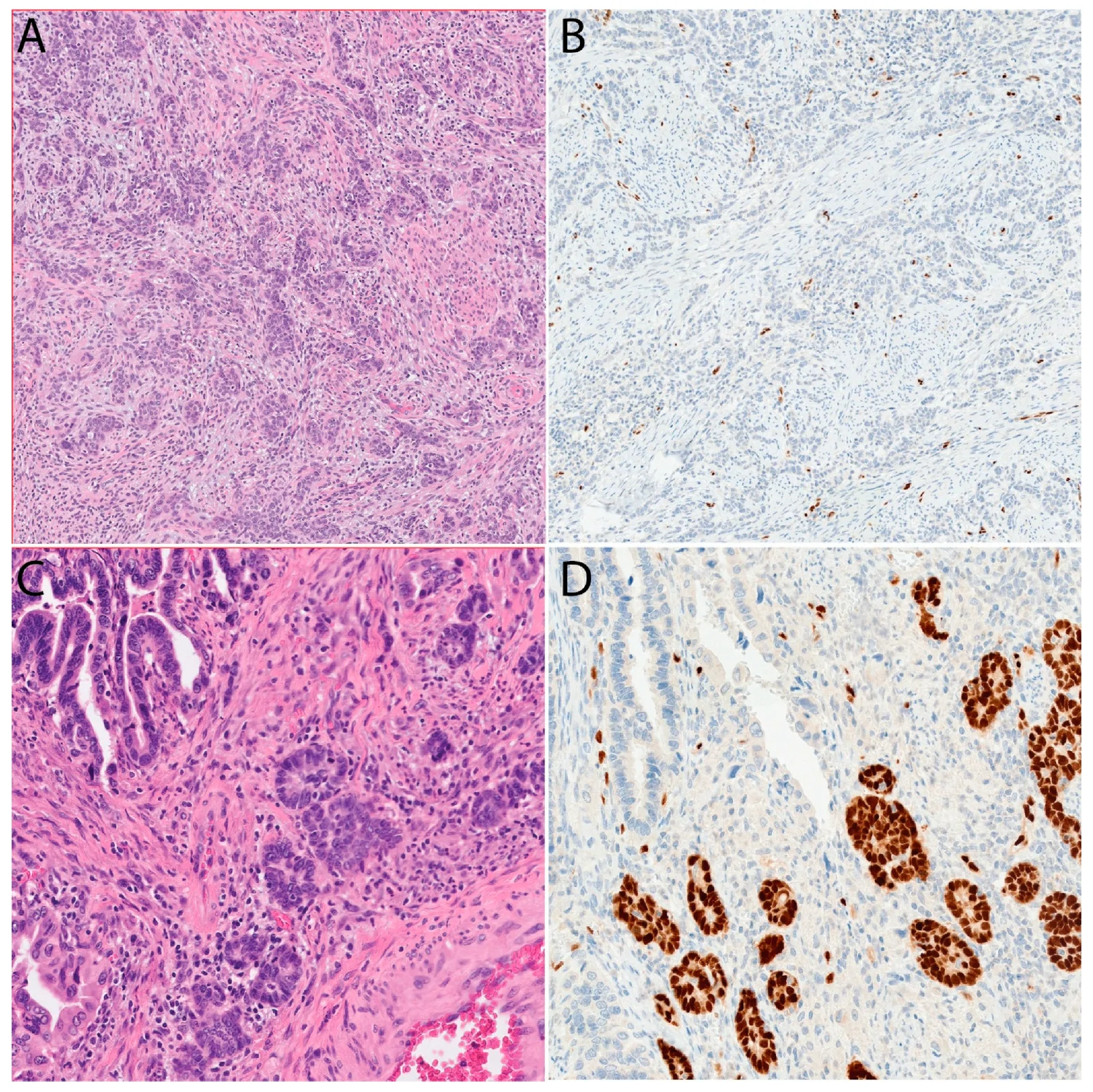
SOX17 expression in uterine cervical tumors. SOX17 immunostaining showed negative result for nuclear staining in cervical squamous cell carcinoma (**A**,**B**), but positive result for nuclear staining in cervical adenocarcinoma (**C**,**D**). (**A**,**C**) Hematoxylin and eosin staining. (**B**,**D**) SOX17 immunostaining. Original magnification, ×100 (**A**,**B**) and ×200 (**C**,**D**).

SOX17 is a developmental transcription factor that demonstrates a highly lineage-restricted expression pattern in the normal uterine cervix. In non-neoplastic cervical tissue, SOX17 is strongly expressed in endocervical columnar epithelium and reserve cells, whereas squamous epithelium is negative. Unlike its consistent expression in the endometrial and ovarian non-mucinous epithelial tumors, SOX17 expression is variable in uterine endocervical adenocarcinomas and completely lost in cervical squamous cell carcinomas [24,25,27,57,58,59]. (Table 2; Figure 3).

In HPV-associated adenocarcinoma in situ (AIS), SOX17 expression is significantly reduced compared with normal endocervical glands. Wang et al. reported SOX17 positivity in only 40.9% of AIS lesions compared with 98.1% of normal endocervical glands [57]. Moreover, SOX17 expression differed substantially among AIS variants. Usual-type AIS retained SOX17 expression in approximately half of cases (51.6%), whereas stratified mucin-producing intraepithelial lesion (SMILE) completely lacked SOX17 expression and AIS with intestinal differentiation showed positivity in only 9.1% of cases [57]. The biological basis for SOX17 loss in AIS and cervical adenocarcinoma appears to be largely epigenetic. Hopman and colleagues demonstrated a strong correlation between SOX17 promoter CpG island hypermethylation and loss of SOX17 protein expression in AIS and invasive adenocarcinoma [58]. Approximately half of glandular neoplasms lacked SOX17 expression, and this loss closely matched promoter methylation status. Notably, SOX17 methylation appeared independent of HPV genotype and viral integration status, indicating that epigenetic silencing represents a distinct molecular event during cervical glandular neoplastic progression [58].

In contrast to cervical glandular lesions, cervical squamous neoplasia consistently demonstrates loss of SOX17 expression. Shaker et al. evaluated 80 cervical carcinomas and found SOX17 expression in 15% of cervical adenocarcinomas and in none of the squamous cell carcinomas. Whole-section analysis gave a higher rate, with focal expression in 40% of adenocarcinomas, and again no staining in squamous carcinomas [24]. Zhang et al. found that SOX17 was expressed at low levels in cervical adenocarcinoma (20%) and had no expression in cervical squamous carcinoma [25]. Moshi et al. found that SOX17-positive immature metaplastic cells and reserve cells gradually lose SOX17 expression during progression toward squamous intraepithelial lesions (SIL) [59]. High-grade squamous intraepithelial lesions (HSIL) and invasive squamous cell carcinomas showed predominantly SOX2-positive/SOX17-negative phenotypes [59]. This SOX17-to-SOX2 switch appears closely associated with squamous differentiation. SOX2 shows the opposite pattern, creating a mutually exclusive SOX17/SOX2 expression profile at the squamocolumnar junction.

Taken together, the available evidence suggests that SOX17 functions as a lineage-associated transcription factor and possibly a tumor suppressor in the uterine cervix. Loss of SOX17 through epigenetic silencing is common during the development of cervical glandular neoplasia, whereas complete absence of SOX17 characterizes most squamous lesions. Assessment of SOX17, especially when combined with SOX2, may provide valuable information regarding tumor lineage, precursor lesion classification, and the molecular pathways driving cervical carcinogenesis.

### 4.3. Germ Cell Tumors

**Figure 4:**
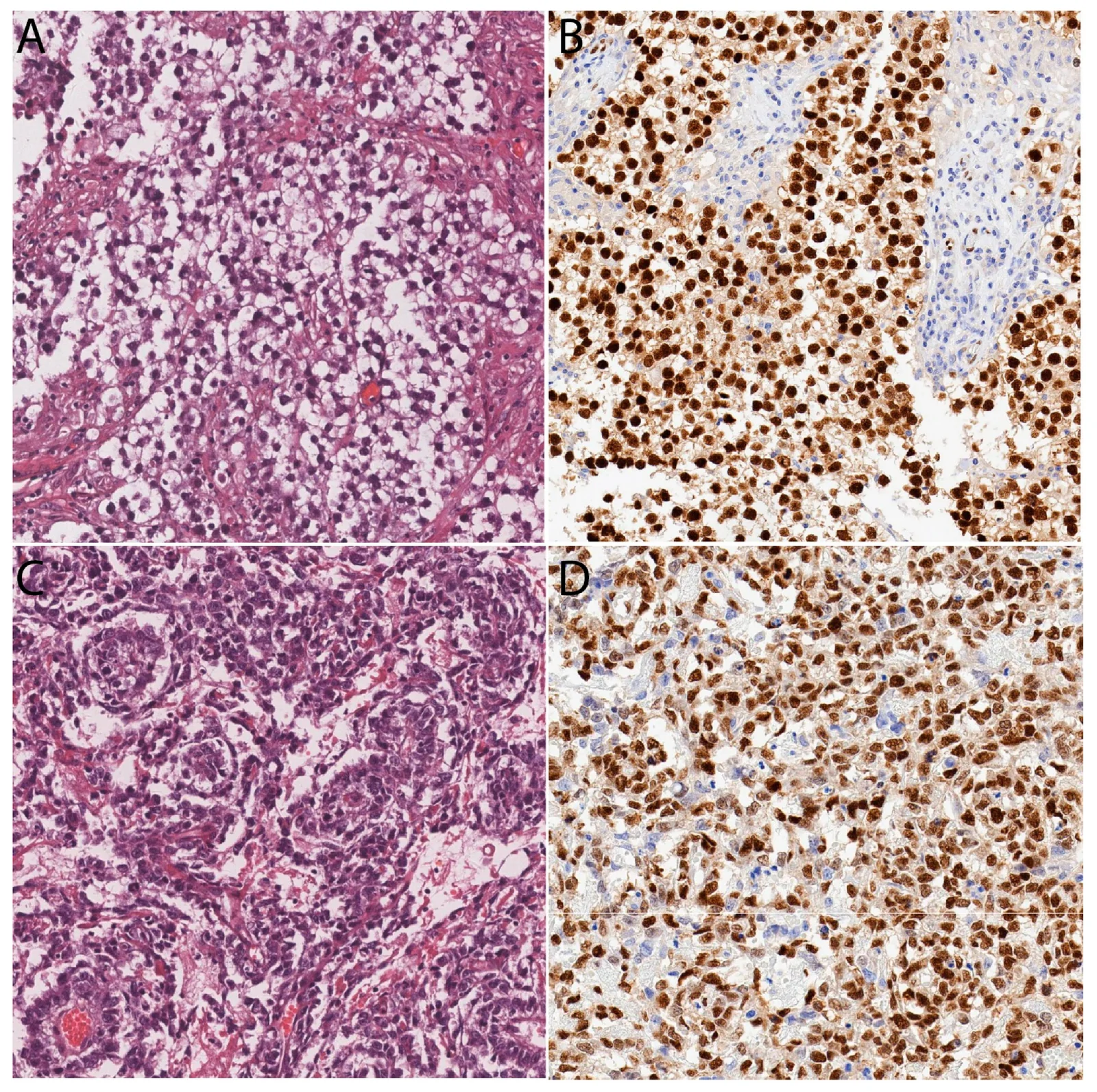
SOX17 expression in germ cell tumors. SOX17 immunostaining showed positive results for nuclear staining in seminoma (**A**,**B**) and yolk sac tumor (**C**,**D**). (**A**,**C**) Hematoxylin and eosin staining. (**B**,**D**) SOX17 immunostaining. Original magnification, ×200.

SOX17 is one of the most important lineage-associated transcription factors in germ cell tumor biology and has been recognized as an immunohistochemical marker for seminomatous germ cell differentiation. Developmental studies have demonstrated that SOX17 is a critical regulator of human primordial germ cell fate [16] and is retained in germ cell neoplasia in situ (GCNIS) and seminomas, supporting the concept that seminomas closely resemble primordial germ cells at both molecular and phenotypic levels [60]. (Table 3; Figure 4).

The study by Nonaka demonstrated that, in a cohort comprising pure and mixed germ cell tumors, all seminoma components exhibited diffuse nuclear SOX17 expression, whereas all embryonal carcinomas lacked SOX17 expression and instead showed diffuse SOX2 positivity [61]. This mutually exclusive staining pattern established SOX17 as a valuable marker for distinguishing seminoma from embryonal carcinoma, particularly in diagnostically challenging cases. In addition, all GCNIS lesions (termed intratubular germ cell neoplasia at the time) showed strong SOX17 expression, further emphasizing the association of SOX17 with the seminomatous developmental pathway [61]. Subsequent studies have confirmed the high sensitivity of SOX17 in seminomatous tumors arising at both gonadal and extragonadal sites. Weissferdt and colleagues examined 32 primary mediastinal seminomas and found SOX17 positivity in 97% of cases, exceeding or matching the sensitivity of established markers such as OCT3/4 and SALL4 [62].

Although SOX17 is most strongly associated with seminoma, its expression is not entirely restricted to this subtype. Nonaka observed variable SOX17 expression in all yolk sac tumors examined, while embryonal carcinomas and choriocarcinomas remained consistently negative [61]. More recently, Zhou et al. reported SOX17 positivity in 96% of seminomas and 73% of yolk sac tumors but no expression in embryonal carcinoma or choriocarcinoma [63]. Limited focal staining was also observed in a subset of teratomas, typically restricted to epithelial components. These findings indicate that while SOX17 is highly sensitive for seminoma, it should not be used as a standalone marker to distinguish seminoma from yolk sac tumor, particularly in mixed germ cell tumors. Instead, SOX17 should be interpreted in conjunction with morphology and complementary markers such as OCT3/4, SOX2, AFP, FOXA2, and glypican-3.

In addition to testicular germ cell tumors, a study by Shaker et al. evaluating SOX17 expression across a broad spectrum of neoplasms identified significant expression in ovarian germ cell tumors [24]. SOX17 was expressed in 75% of ovarian dysgerminomas and 100% of yolk sac tumors, whereas ovarian sex cord-stromal tumors were consistently negative. These findings parallel observations in testicular germ cell tumors and suggest that SOX17 may be useful in the diagnostic evaluation of ovarian germ cell neoplasms.

Taken together, the available evidence supports SOX17 as a useful marker of seminomatous germ cell differentiation. Its high sensitivity for seminoma and dysgerminoma, lack of expression in embryonal carcinoma, and strong correlation with primordial germ cell biology make it a valuable diagnostic and biologic marker. However, its expression in yolk sac tumors limits specificity, necessitating interpretation within a broader immunohistochemical panel. Current evidence supports the combined use of SOX17, SOX2, OCT3/4, and SALL4 as a practical approach to classification of germ cell tumor subtypes.

### 4.4. Vascular Tumors

**Figure 5:**
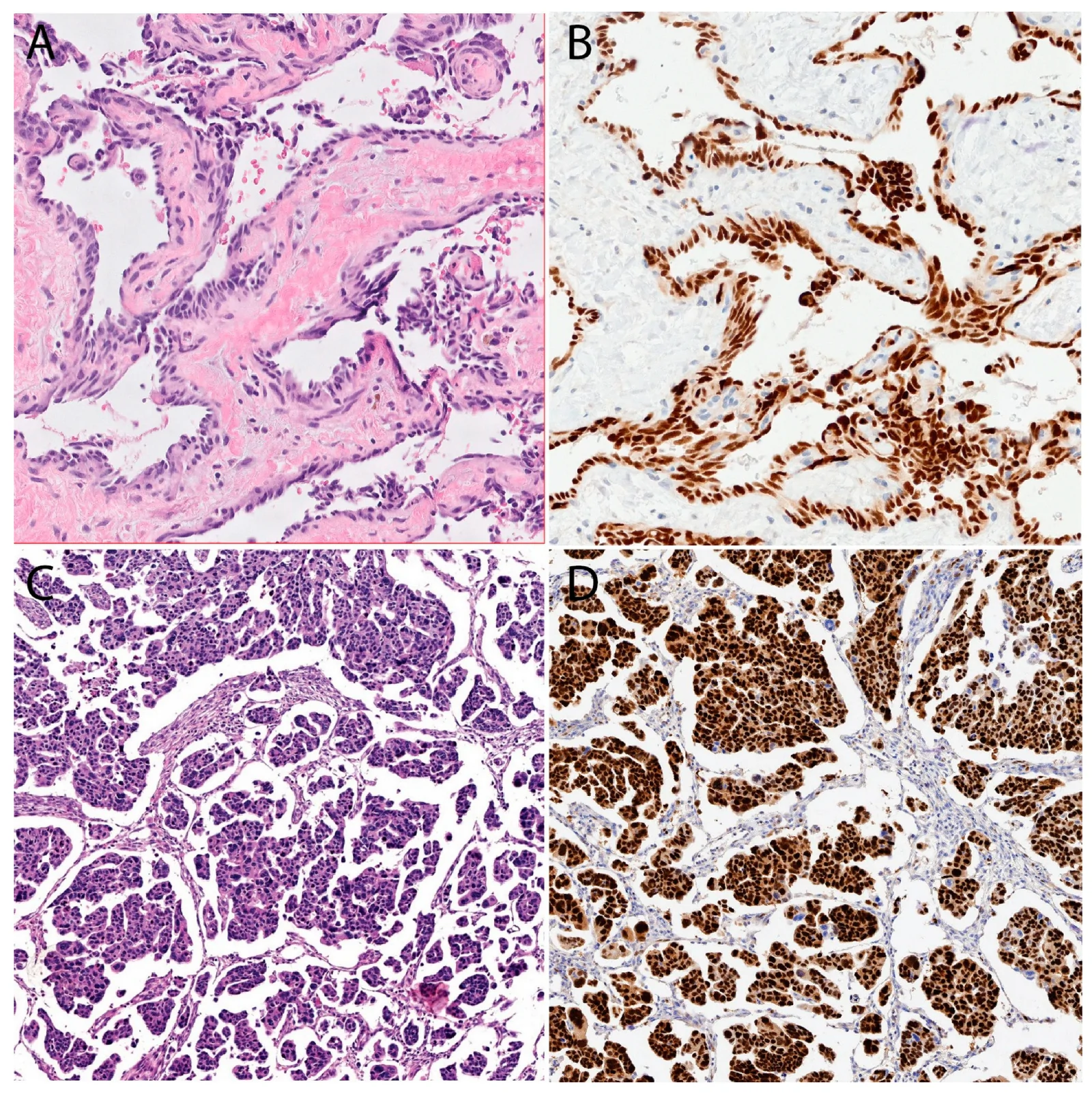
SOX17 expression in vascular tumor and mesothelioma. SOX17 immunostaining showed positive results for nuclear staining in angiosarcoma (**A**,**B**) and mesothelioma (**C**,**D**). (**A**,**C**) Hematoxylin and eosin staining. (**B**,**D**) SOX17 immunostaining. Original magnification, ×100.

Beyond its established role as a marker of Müllerian carcinoma, accumulating evidence demonstrates that SOX17 is physiologically expressed in vascular endothelial cells and may serve as a useful marker of endothelial differentiation. (Table 4; Figure 5A,B).

Shaker et al. demonstrated diffuse nuclear SOX17 expression in normal vascular endothelial cells [24]. The biologic significance of SOX17 expression in endothelial cells is supported by prior developmental studies demonstrating essential roles in cardiovascular development, endothelial differentiation, and angiogenic regulation. Experimental evidence has shown that SOX17 promotes angiogenesis and participates in vascular remodeling [64,65]. Furthermore, in ovarian carcinoma, SOX17 has been implicated in angiogenic signaling pathways through interaction with PAX8, suggesting that SOX17 expression may be linked not only to endothelial differentiation but also to active vascular remodeling and tumor-associated angiogenesis [46].

**Table 4:** SOX17 Expression in Normal Endothelial Cells and Vascular Tumors.

Study	Tissue/Tumor Type	Cases (n)	SOX17 Positivity	Key Findings
Shaker et al. 2023 [24]	Normal vascular endothelial cells	25	100% (25/25)	Diffuse nuclear staining in endothelial cells across multiple tissues; useful internal positive control.
Shaker et al. 2023 [24]	Angiosarcoma	10	100% (10/10)	All cases positive; 8 diffusely positive and 2 intermediately positive.
Ibrahim et al. (USCAP 2026) [66]	Angiosarcoma	34	88.2% (30/34)	Predominantly strong and diffuse staining (>80% tumor cells).
Ibrahim et al. (USCAP 2026) [66]	Epithelioid hemangioendothelioma (EHE)	14	7.1% (1/14)	Only one case showed focal weak staining; 92.9% negative.

Abbreviations: EHE, epithelioid hemangioendothelioma; SOX17, SRY-box transcription factor 17; USCAP, United States and Canadian Academy of Pathology.

The first substantial evaluation of SOX17 in malignant vascular tumors was reported by Shaker et al., who examined 10 angiosarcomas. All angiosarcomas demonstrated nuclear SOX17 positivity, with eight cases showing diffuse (>50%) staining and two cases showing intermediate positivity [24]. The authors noted that SOX17 expression was present in both well-differentiated and poorly differentiated angiosarcomas, suggesting that expression is maintained throughout the spectrum of angiosarcoma differentiation. These findings also highlighted a potential advantage over PAX8, which is typically negative in angiosarcomas.

More recently, Ibrahim et al. expanded the evaluation of SOX17 expression in vascular neoplasms through a study of 34 angiosarcomas and 14 epithelioid hemangioendotheliomas (EHE) [66]. SOX17 nuclear positivity was observed in 88.2% (30/34) of angiosarcomas, with staining typically diffuse, involving greater than 80% of tumor cells, and of strong intensity. In contrast, only a single EHE case (7.1%) demonstrated focal weak staining [66]. The difference in expression between angiosarcoma and EHE was highly significant, suggesting that SOX17 may aid in distinguishing these entities in diagnostically difficult cases, especially when biopsy material is limited. The differential expression of SOX17 between angiosarcoma and EHE may also provide insight into tumor pathogenesis. Angiosarcoma is characterized by aggressive endothelial proliferation and active angiogenesis, processes in which SOX17 is known to be involved. In contrast, EHE is driven by WWTR1::CAMTA1 or, less often, YAP1::TFE3 fusions and lacks the highly angiogenic phenotype seen in angiosarcoma. The marked difference in SOX17 expression between these tumors therefore appears to reflect fundamental biologic distinctions rather than merely lineage-related differentiation. From a practical diagnostic perspective, SOX17 should be viewed as a potentially useful adjunctive vascular marker rather than a replacement for traditional endothelial markers. ERG, CD31, and CD34 remain highly sensitive markers for endothelial differentiation, but all are expressed in both angiosarcoma and EHE. SOX17 differs in that it demonstrates preferential expression in angiosarcoma while remaining negative or only rarely positive in EHE. Thus, incorporation of SOX17 into immunohistochemical panels may improve diagnostic accuracy when evaluating malignant vascular neoplasms, particularly in the distinction between angiosarcoma and EHE.

Overall, current evidence supports SOX17 as both a physiologic marker of normal endothelial cells and a highly sensitive marker of angiosarcoma. Its expression reflects its central role in endothelial differentiation and angiogenesis, and emerging data suggest that SOX17 may have significant diagnostic utility in vascular tumor pathology. Additional studies involving larger cohorts and broader spectra of vascular neoplasms will be valuable in further defining the sensitivity and specificity of SOX17 in this setting.

### 4.5. Mesothelial Proliferations

The potential diagnostic value of SOX17 stems from the well-recognized challenge of differentiating mesothelial proliferations from Müllerian neoplasms. Malignant peritoneal mesothelioma, well-differentiated papillary mesothelial tumor (WDPMT), adenomatoid tumor, and peritoneal inclusion cysts may all mimic ovarian serous neoplasms clinically, radiologically, and histologically. Existing markers such as WT1, calretinin, and D2-40 show substantial overlap between mesothelial and serous Müllerian lesions. Furthermore, PAX8, traditionally considered a marker of Müllerian differentiation, has been reported in a subset of peritoneal mesotheliomas and benign mesothelial proliferations, limiting its specificity (Table 5; Figure 5C,D).

In the study by Zhang et al., SOX17 expression was evaluated in 35 mesotheliomas, including thoracic, abdominal, and pelvic tumors, and all cases were completely negative [25]. A subsequent study by Laokulrath et al. systematically investigated SOX17 expression in 20 benign mesothelial proliferations and 16 epithelioid peritoneal mesotheliomas [67]. Contrary to earlier reports, SOX17 positivity was identified in 25% of benign mesothelial lesions and 13% of mesotheliomas. Among benign lesions, staining was observed in adenomatoid tumors and peritoneal inclusion cysts, whereas all WDPMTs were negative. In mesotheliomas, two cases demonstrated SOX17 positivity, including one case with diffuse strong nuclear staining involving more than 90% of tumor cells [67]. These findings demonstrated that SOX17 positivity cannot be regarded as entirely specific for Müllerian carcinomas and highlighted the risk of diagnostic misclassification when SOX17 is used in isolation.

More recently, Li et al. expanded the investigation substantially by examining 124 malignant mesotheliomas, 20 normal mesothelial samples, 20 adenomatoid tumors, 22 peritoneal inclusion cysts, 12 WDPMTs, and 39 cytologic specimens containing benign mesothelial cells [51]. Their study confirmed that SOX17 expression occurs in a subset of mesothelial proliferations. SOX17 positivity was identified in 16.9% of mesotheliomas, with most positive cases showing diffuse staining involving more than 50% of tumor cells. Interestingly, expression was significantly more common in women and in peritoneal mesotheliomas than in pleural or pericardial tumors. Among benign mesothelial lesions, SOX17 was expressed in 33.3% of WDPMTs and 9.1% of peritoneal inclusion cysts but was absent in all adenomatoid tumors. Most notably, SOX17 expression was completely absent in normal mesothelium and benign mesothelial cells present in pleural and peritoneal fluid specimens.

Collectively, the available literature suggests that SOX17 expression is not a feature of normal mesothelial cells but may emerge in a subset of benign and malignant mesothelial proliferations. The prevalence of SOX17 positivity appears variable across studies, ranging from 0% to nearly 17% in mesotheliomas. Differences in patient populations, tumor site distribution, sample size, and use of tissue microarrays versus whole tissue sections likely contribute to the discrepancies. Notably, both studies reporting positive staining found a predominance among female patients and peritoneal lesions, suggesting the possibility of biologic heterogeneity among mesotheliomas [51,67].

From a diagnostic standpoint, pathologists should be aware that a subset of mesotheliomas, WDPMTs, adenomatoid tumors, and peritoneal inclusion cysts may also express this marker. Accordingly, SOX17 should be incorporated into a broader immunohistochemical panel that includes mesothelial markers (calretinin, WT1, D2-40), epithelial markers (claudin-4, BerEP4), and Müllerian markers (PAX8, ER) together with clinicopathologic correlation. The absence of SOX17 in normal mesothelium may also suggest that SOX17 activation is associated with mesothelial proliferation or neoplastic transformation rather than normal mesothelial differentiation.

### 4.6. SOX17 Antibody Clones for Diagnostic Immunohistochemistry

Because antibody clone selection directly affects staining sensitivity and reproducibility, we summarize the SOX17 antibody clones most frequently reported in the diagnostic gynecologic pathology literature (Table 6). The rabbit recombinant monoclonal clone EPR20684 (Abcam) has been used in the principal validation studies establishing SOX17 as a Müllerian lineage marker and is, to our knowledge, the clone most widely adopted by clinical immunohistochemistry laboratories; other commercially available clones and polyclonal reagents exist but have limited independent validation in large diagnostic cohorts.

As with any immunohistochemical biomarker, individual laboratories must independently validate the specific antibody clone, dilution, and detection platform used in their practice against appropriate positive (endothelial cells, fallopian tube/endometrial epithelium) and negative controls before clinical implementation, consistent with College of American Pathologists and other applicable laboratory-developed test validation requirements; reported sensitivity and specificity figures cited throughout this review are clone- and protocol-specific and may not be directly transferable across reagents.

## 5. Comparison of SOX17 and PAX8 in Diagnostic Evaluation of Tumors

PAX8 has long served as the principal immunohistochemical marker used to establish Müllerian, renal, and thyroid lineage; however, its relatively broad expression profile creates significant diagnostic challenges in the differential diagnosis of metastatic carcinomas. In contrast, several recent studies have demonstrated that SOX17 exhibits a staining pattern that largely parallels PAX8 in ovarian and endometrial carcinomas while showing substantially greater specificity in most non-Müllerian tumors. Consequently, SOX17 has emerged as a potentially valuable adjunct or alternative marker for determining gynecologic origin (Table 7).

The largest comparative studies by Zhang et al. and Shaker et al. demonstrated that SOX17 and PAX8 have similar sensitivities in most Müllerian epithelial malignancies [24,25]. In ovarian serous carcinoma, SOX17 and PAX8 showed nearly identical positivity rates (97.5% vs 97%, respectively), while both markers were highly expressed in ovarian endometrioid carcinomas (90% each) and ovarian clear cell carcinomas (100% each). Similarly, in endometrial carcinoma, SOX17 exhibited sensitivity equivalent to or slightly greater than PAX8, with positivity in 88% versus 84% of endometrioid carcinomas and 100% positivity in serous and clear cell carcinomas. In most cases, co-expression was observed, further supporting their shared biologic relationship within Müllerian epithelium.

Although the overall expression of SOX17 and PAX8 is comparable in Müllerian neoplasms, important differences emerge in specific histologic subtypes. MLAs represent the clearest example. Recent studies demonstrated that most MLAs exhibit loss or decreased expression of SOX17 while maintaining diffuse strong PAX8 expression [53,54,55]. This supports incorporating SOX17 into a multiparametric immunohistochemical approach to improve the diagnostic accuracy of MLA [56]. Mucinous ovarian carcinomas represent another example. SOX17 expression is largely absent in ovarian mucinous carcinomas, whereas PAX8 remains positive in approximately 18–27% of cases. Likewise, SOX17 expression may be reduced or lost in dedifferentiated and undifferentiated endometrial carcinomas, high-grade neuroendocrine carcinomas, and some cervical adenocarcinomas. Therefore, although SOX17 is highly sensitive for the major Müllerian carcinoma types, PAX8 may retain a slight advantage in selected uncommon Müllerian tumors.

The greatest diagnostic advantage of SOX17 over PAX8 lies in its superior specificity. Whereas PAX8 is strongly expressed in thyroid and renal neoplasms, SOX17 was negative in the renal and thyroid cohorts examined. In the study by Zhang et al., PAX8 was positive in 86% of thyroid carcinomas and 85% of renal carcinomas, while SOX17 was negative in 100% of both groups [25]. Similar findings were reported by Shaker et al., who found complete absence of SOX17 expression in 110 renal tumors and 70 thyroid tumors [24]. These observations are clinically important because thyroid and renal carcinomas frequently enter the differential diagnosis of metastatic Müllerian tumors, particularly when poorly differentiated or presenting at distant sites.

Additional studies have further demonstrated the high specificity of SOX17 in tumors from other organ systems. SOX17 is negative, with rare exceptions, in breast carcinoma, lung carcinoma, colorectal carcinoma, pancreatic adenocarcinoma, urothelial carcinoma, salivary gland carcinoma, hepatocellular carcinoma, and most cholangiocarcinomas, whereas PAX8 may occasionally be expressed in some of these tumors, particularly breast and urothelial carcinomas [24,25]. Clark et al. examined more than 1000 neoplasms and found SOX17 expression almost exclusively restricted to ovarian, uterine, and testicular tumors [27]. Likewise, Zhang et al. observed complete absence of SOX17 staining in more than 1500 nongynecologic tumors, including breast, lung, colon, stomach, pancreas, bladder, liver, and mesothelioma [25].

Another important distinction between the two markers involves mesothelial lesions. PAX8 expression has been reported in a subset of malignant peritoneal mesotheliomas, benign mesothelium, and other mesothelial proliferations, creating a well-recognized diagnostic pitfall. Initial studies suggested complete absence of SOX17 in mesothelioma, supporting its superiority over PAX8 in this setting [25]. More recent studies, however, have shown that approximately 13–17% of mesotheliomas may express SOX17 [51,67]. Nevertheless, SOX17 remains less frequently expressed in mesothelial lesions than PAX8 and therefore generally retains greater specificity for Müllerian differentiation.

A unique feature of SOX17 is its expression in endothelial cells and vascular neoplasms. Unlike PAX8, SOX17 is consistently expressed in normal endothelial cells and demonstrates high sensitivity for angiosarcoma. Shaker et al. reported SOX17 positivity in all tested angiosarcomas, while a subsequent study reported positivity in approximately 88% of cases [24]. This endothelial expression pattern serves as a useful internal positive control during immunohistochemical interpretation but also represents a potential diagnostic pitfall when evaluating vascular tumors.

Emerging evidence also indicates that SOX17 may have utility in germ cell tumors. Both ovarian dysgerminomas and testicular seminomas frequently express SOX17, whereas PAX8 is generally negative [24,61]. This broader expression in germ cell neoplasia reflects the biologic role of SOX17 in primordial germ cell development and further distinguishes its staining profile from that of PAX8.

Overall, contemporary evidence suggests that SOX17 and PAX8 possess similar sensitivity for most ovarian and endometrial carcinomas, but SOX17 demonstrates substantially greater specificity because it lacks expression in most renal, thyroid, breast, pulmonary, gastrointestinal, pancreatic, and urothelial tumors. Consequently, SOX17 may be particularly valuable when the differential diagnosis includes PAX8-positive non-Müllerian neoplasms. The most effective diagnostic strategy is likely the combined use of SOX17 and PAX8, which exploits the high sensitivity of both markers while improving overall specificity for Müllerian origin.

## 6. Biologic and Therapeutic Implications in Gynecologic Oncology

**Figure 6:**
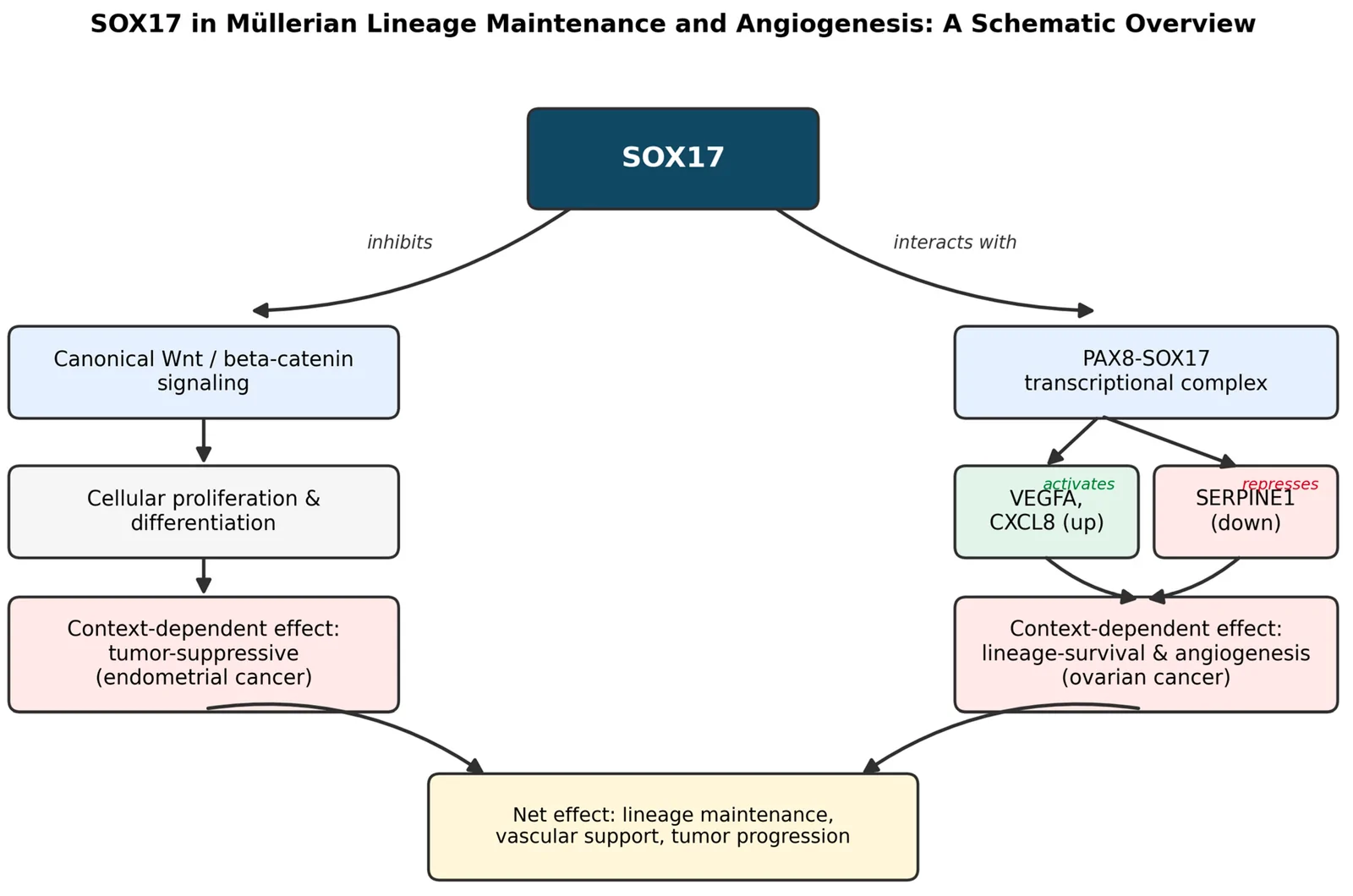
Schematic overview of SOX17 mechanistic activity in gynecologic tissues (original diagram; not previously published). SOX17 modulates two partially distinct, context-dependent programs: inhibition of canonical Wnt/β-catenin signaling, which behaves in a tumor-suppressive manner in endometrial carcinoma, and formation of a PAX8-SOX17 transcriptional complex that upregulates pro-angiogenic mediators (VEGFA, CXCL8) and represses SERPINE1, supporting lineage survival and vascular maintenance in ovarian carcinoma. Both arms converge on a net phenotype of Müllerian lineage maintenance and, in the malignant context, tumor progression.

Beyond its diagnostic utility (Section 4), SOX17 exerts biologic effects that are directly relevant to gynecologic oncology practice (Figure 6).

SOX17 serves as a transcriptional node integrating epithelial differentiation, angiogenesis, and cell-cycle control. The cooperative transcriptional network formed by SOX17 and PAX8 underlies key processes in ovarian and endometrial carcinoma, particularly angiogenesis and lineage survival, revealing potential therapeutic targets within this axis. Recent studies demonstrate that SOX17 directly interacts with PAX8 to activate pro-angiogenic mediators such as VEGFA and CXCL8, while repressing anti-angiogenic factors including SERPINE1 [46]. Disruption of this PAX8–SOX17 axis or its vascular downstream effectors may represent a lineage-targeted therapeutic strategy that selectively impairs tumor growth while sparing normal Müllerian tissues [45]. In endometrial carcinoma, SOX17 expression is associated with favorable clinicopathologic features, including low grade, early stage, and absence of nodal or distant metastasis, whereas loss of expression correlates with recurrence and reduced overall and disease-free survival [68].

Future research should focus on defining the SOX17 interactome and downstream signaling pathways across histologic subtypes, exploring its predictive relevance in targeted and immune therapies, and leveraging its stable nuclear expression for quantitative digital pathology applications. The PAX8-SOX17 axis is the most concrete translational lead to come out of this work.

## 7. Conclusions

This review makes three principal contributions to the SOX17 literature in gynecologic pathology. First, it consolidates evidence across normal tissues, epithelial and non-epithelial neoplasms, and diagnostic pitfalls into a single lineage-based framework, clarifying where SOX17 adds genuine diagnostic value beyond PAX8 (particularly its superior specificity for Müllerian origin) and where its expression instead demands cautious interpretation, as in seminomatous germ cell tumors, yolk sac tumors, vascular neoplasms, and subsets of mesothelial proliferations. Second, it translates this evidence into practical diagnostic guidance, including a summary of the antibody clones used in published validation studies (Table 6) to support laboratories implementing or troubleshooting SOX17 immunohistochemistry. Third, it identifies concrete, testable gaps for future work, most notably the absence of studies systematically correlating SOX17 expression with the TCGA/WHO molecular subtypes of endometrial carcinoma (POLE-mutant, mismatch repair-deficient, p53-abnormal, and NSMP) and the incompletely defined mechanistic basis of the SOX17-PAX8 transcriptional axis in angiogenesis and lineage survival. SOX17 has evolved from a developmental transcription factor into one of the most informative lineage-associated biomarkers in modern surgical pathology. Its highly restricted expression in Müllerian-derived epithelia, coupled with exceptional specificity for ovarian and endometrial carcinomas, provides significant diagnostic advantages over traditional lineage markers, particularly in the evaluation of metastatic disease and limited tissue or cytology specimens. At the same time, recognition of its expression in endothelial cells, seminomatous germ cell tumors, yolk sac tumors, a minority of cervical adenocarcinomas, and subsets of mesothelial proliferations underscores the importance of interpreting SOX17 within an appropriate morphologic and immunophenotypic context. Beyond diagnosis, growing mechanistic evidence identifies SOX17 as a context-dependent regulator of lineage identity, angiogenesis, and tumor progression through interactions with signaling pathways such as Wnt/β-catenin and the PAX8 transcriptional network. These biological insights expand the significance of SOX17 beyond immunohistochemistry, highlighting its potential as a prognostic biomarker, therapeutic target, and quantitative digital pathology marker. Looking forward, we see three priorities for the field: (1) correlative studies integrating SOX17 immunohistochemistry with POLE sequencing, mismatch repair testing, and p53 immunostaining to determine whether SOX17 expression tracks with, or adds prognostic information within, specific molecular subtypes of endometrial carcinoma; (2) multi-institutional reproducibility studies comparing the diagnostic performance of different SOX17 antibody clones and staining platforms; and (3) mechanistic studies clarifying whether disruption of the SOX17-PAX8 axis or its downstream angiogenic effectors can be therapeutically exploited without compromising normal Müllerian tissue homeostasis. Continued investigation along these lines will further define the role of SOX17 in precision oncology and strengthen its integration into future diagnostic and translational pathology practice.

## Figures and Tables

**Table 1:** SOX17 Expression in Endometrial and Ovarian Epithelial Tumors.

Study	Cohort	Major Findings in Ovarian/Endometrial Carcinomas	Sensitivity	Specificity/Diagnostic Pitfalls
Shaker et al. 2023 [24]	398 ovarian tumors, 93 endometrial carcinomas, 1371 nongynecologic tumors	Strong expression in serous, clear cell, and endometrioid ovarian carcinomas and endometrial carcinomas; absent or reduced in mucinous tumors	Ovarian serous 91%, clear cell 91%, endometrioid 74%; endometrial carcinoma 89%	Negative in thyroid, kidney, breast, colon, pancreas, lung, melanoma; positive in germ cell tumors and angiosarcoma
Zhang et al. 2023 [25]	327 ovarian and 89 endometrial carcinomas; 1544 nongynecologic tumors	Comparable sensitivity to PAX8; highly expressed in serous, endometrioid, and clear cell carcinomas	Ovarian serous 97.5%, endometrioid 90%, clear cell 100%; endometrial endometrioid 88%, serous/clear cell 100%	Negative in renal and thyroid carcinomas
Satturwar et al. 2023 [26]	29 metastatic gynecologic carcinomas, 55 nongynecologic carcinomas (cytology cell blocks)	Diffuse strong nuclear staining in all metastatic ovarian and endometrial carcinomas	100%	98.2%; one papillary thyroid carcinoma showed focal positivity (<10%)
Shrestha et al. 2025 [52]	30 metastatic gynecologic tumors and 55 nongynecologic tumors in effusions	All gynecologic tumors positive; most showed diffuse strong staining	100%	One renal cell carcinoma showed patchy positivity; all thyroid tumors negative
Clark et al. 2025 [27]	1004 tumors from 20 organ sites	SOX17 positive in 94% of endometrial tumors and 96% of ovarian tumors; expression reduced in mucinous and dedifferentiated tumors	Endometrial 94%; ovarian 96%	Negative in all breast tumors and virtually all nongynecologic carcinomas
Moustafa et al. 2026 [28]	56 endometrial, 56 ovarian, 56 nongynecologic tumors	PAX8-high/SOX17-high phenotype strongly associated with endometrioid tumors	High SOX17 expression in most Müllerian tumors	Double-negative SOX17/PAX8 excluded Müllerian origin in colorectal, breast, and pulmonary carcinomas

Abbreviations: PAX8, paired box 8; SOX17, SRY-box transcription factor 17.

**Table 2:** SOX17 Expression in Uterine Cervical Neoplasms.

Study	Lesions Examined	SOX17 Expression	Major Findings
Hopman et al. 2020 [58]	23 AIS, 22 invasive adenocarcinomas	Approximately 50% positive; loss correlated with promoter methylation	Promoter hypermethylation strongly associated with loss of SOX17 expression
Moshi et al. 2020 [59]	LSIL, HSIL, SCC, AIS	Predominantly lost in SIL and SCC	SOX17-to-SOX2 switch during progression from reserve cells/immature metaplasia to squamous dysplasia.
Wang et al. 2026 [57]	159 HPV-associated AIS (124 usual-type, 24 SMILE, 11 intestinal-type)	Overall AIS: 40.9%; usual-type AIS: 51.6%; SMILE: 0%; intestinal-type AIS: 9.1%	Significant reduction compared with normal endocervical glands (98.1%). SOX2+/SOX17− profile highly characteristic of SMILE.
Shaker et al. 2023 [24]	40 cervical adenocarcinomas, 40 squamous cell carcinomas (TMA); additional whole sections	Adenocarcinoma: 15% positive (TMA), 40% positive (whole sections); SCC: 0% positive	Most cervical adenocarcinomas and all squamous carcinomas lacked SOX17 expression.
Zhang et al. 2023 [25]	25 cervical adenocarcinomas, 115 squamous cell carcinomas	Adenocarcinoma: 20% positive; SCC: 0% positive	Most cervical adenocarcinomas and all squamous carcinomas lacked SOX17 expression.

Abbreviations: AIS, adenocarcinoma in situ; HPV, human papillomavirus; HSIL, high-grade squamous intraepithelial lesion; LSIL, low-grade squamous intraepithelial lesion; SCC, squamous cell carcinoma; SMILE, stratified mucin-producing intraepithelial lesion; SOX2, SRY-box transcription factor 2; SOX17, SRY-box transcription factor 17; TMA, tissue microarray.

**Table 3:** SOX17 Expression in Germ Cell Tumors.

Study	Tumor Type	Cases (n)	SOX17 Positivity	Major Findings
Nonaka, 2009 [61]	Testicular seminoma	41	100%	All seminomas showed diffuse nuclear SOX17 expression; SOX17 absent in embryonal carcinoma.
Nonaka, 2009 [61]	Embryonal carcinoma	30	0%	Complete absence of SOX17; reciprocal SOX2 positivity.
Nonaka, 2009 [61]	Yolk sac tumor	17	100% (variable intensity)	Variable but consistent SOX17 expression.
Nonaka, 2009 [61]	Teratoma	19	42%	Restricted to epithelial elements.
Nonaka, 2009 [61]	Choriocarcinoma	5	0%	Completely negative.
Nonaka, 2009 [61]	GCNIS	Not specified	100%	Strong diffuse staining highlighting preinvasive lesions.
Weissferdt et al. 2015 [62]	Primary mediastinal seminoma	32	97% (31/32)	Strong diffuse nuclear staining; highly sensitive marker for extragonadal seminoma.
Zhou et al. 2022 [63]	Seminoma (pure and mixed)	25	96% (24/25)	Sensitivity comparable to PRAME; generally stronger and more diffuse staining.
Zhou et al. 2022 [63]	Embryonal carcinoma	35	0%	Uniformly negative.
Zhou et al. 2022 [63]	Yolk sac tumor	15	73% (11/15)	Frequent positivity; limits specificity for seminoma.
Zhou et al. 2022 [63]	Teratoma	23	17.4%	Focal expression limited to epithelial elements.
Zhou et al. 2022 [63]	Choriocarcinoma	5	0%	Negative in all cases.
Zhou et al. 2022 [63]	GCNIS	11	100%	Diffuse nuclear expression.
Shaker et al. 2023 [24]	Ovarian dysgerminoma	8	75% (6/8)	Supports utility in ovarian seminomatous tumors.
Shaker et al. 2023 [24]	Ovarian yolk sac tumor	7	100% (7/7)	Strong expression in ovarian YSTs.
Shaker et al. 2023 [24]	Ovarian sex cord-stromal tumors	22	0%	Useful distinction from germ cell tumors.

Abbreviations: GCNIS, germ cell neoplasia in situ; PRAME, preferentially expressed antigen in melanoma; SOX2, SRY-box transcription factor 2; SOX17, SRY-box transcription factor 17; YST, yolk sac tumor.

**Table 5:** SOX17 Expression in Mesothelial Tissue and Mesothelial Lesions.

Study	Lesion Type	Cases (n)	SOX17 Positive	Key Findings
Zhang et al. 2023 [25]	Mesothelioma	35	0% (0/35)	All thoracic, abdominal, and pelvic mesotheliomas were negative. Suggested high specificity for Müllerian carcinomas.
Laokulrath et al. 2025 [67]	Mesothelioma (peritoneal)	16	13% (2/16)	One diffusely strong positive case and one focal weak positive case.
Laokulrath et al. 2025 [67]	Benign mesothelial proliferations (overall)	20	25% (5/20)	SOX17 positivity documented in benign mesothelial lesions.
Laokulrath et al. 2025 [67]	Adenomatoid tumor	5	40% (2/5)	Two adenomatoid tumors showed moderate to strong staining.
Laokulrath et al. 2025 [67]	WDPMT	5	0% (0/5)	No SOX17 expression identified.
Laokulrath et al. 2025 [67]	Peritoneal inclusion cyst	10	30% (3/10)	Variable moderate to strong staining observed.
Li et al. 2025 [51]	Mesothelioma (overall)	124	16.9% (21/124)	Majority showed diffuse (>50%) staining; female and peritoneal tumors stained more frequently.
Li et al. 2025 [51]	Peritoneal inclusion cyst	22	9.1% (2/22)	Occasional positivity observed.
Li et al. 2025 [51]	WDPMT	12	33.3% (4/12)	Highest positivity rate among benign mesothelial lesions.
Li et al. 2025 [51]	Adenomatoid tumor	20	0% (0/20)	Completely negative.
Li et al. 2025 [51]	Normal mesothelium	20	0% (0/20)	No SOX17 expression in normal mesothelial lining.
Li et al. 2025 [51]	Benign mesothelial cells in effusions	39	0% (0/39)	No staining in pleural or peritoneal fluid mesothelial cells.

Abbreviations: SOX17, SRY-box transcription factor 17; WDPMT, well-differentiated papillary mesothelial tumor.

**Table 6:** Representative SOX17 Antibody Clones Used in Diagnostic Immunohistochemistry.

Antibody Clone	Host/Format	Representative Commercial Source	Reported IHC Conditions	Diagnostic Validation Status
EPR20684	Rabbit, recombinant monoclonal	Abcam (ab224637); repackaged by several IHC reagent vendors	~1:1000–1:1500 dilution; heat-induced epitope retrieval (EDTA/Tris, pH 9); automated platforms (Leica BOND, Ventana)	Most extensively validated clone in the gynecologic-pathology literature; used in the principal SOX17 sensitivity/specificity studies cited in this review; nuclear staining with endothelial internal positive control
3F7 (ZooMAb)	Rabbit, recombinant monoclonal	Sigma-Aldrich/MilliporeSigma (ZRB1345)	~1:1000 dilution (IHC-P), manufacturer-reported	Alternative recombinant clone; minimally represented in published gynecologic diagnostic validation series
Polyclonal antibodies	Rabbit, polyclonal	Sigma-Aldrich (09-038-I), R&D Systems (AF1924), and others	Variable; manufacturer- and lot-dependent	Used mainly in developmental-biology and research applications; lot-to-lot variability and absence of large diagnostic validation series limit routine clinical use
Research-use monoclonals (e.g., O92B6.Rec, OTI3B10)	Mouse, monoclonal	BioLegend, OriGene, and others	Optimized for Western blot/ICC/flow cytometry; IHC-P performance not systematically reported in tumor cohorts	Labeled for research use only; not validated for diagnostic surgical pathology

**Table 7:** Comparison of SOX17 and PAX8 Expression in Human Tumors.

Tumor Type	SOX17 Expression	PAX8 Expression	Diagnostic Implication
Ovarian serous carcinoma	91–100%; 97.5% in large series	79–99%; 97% in large series	Similar sensitivity for Müllerian origin.
Ovarian endometrioid carcinoma	74–100%; ~90%	38–92%; ~90%	Similar sensitivity.
Ovarian clear cell carcinoma	91–100%	76–100%	Comparable performance.
Ovarian mucinous carcinoma	0–24%	0–50% (often 18–27%)	PAX8 slightly more sensitive; SOX17 usually absent.
Ovarian carcinosarcoma	Predominantly epithelial component positive	Similar pattern	Comparable expression.
Endometrioid endometrial carcinoma	88–100%	84–100%	SOX17 slightly more uniform.
Endometrial serous carcinoma	100%	100%	Equivalent sensitivity.
Endometrial clear cell carcinoma	80–100%	100%	Nearly equivalent.
Endometrial carcinosarcoma	Epithelial component positive	Epithelial component positive	Similar expression patterns.
Cervical adenocarcinoma	15–40%	0–50%	Limited sensitivity for both markers.
Cervical squamous carcinoma	0%	Usually negative	Neither marker particularly useful.
Renal cell carcinoma	0%	85–100%	Major specificity advantage of SOX17.
Thyroid carcinoma	0% (rare focal exceptions)	70–100%	Major specificity advantage of SOX17.
Breast carcinoma	0%	6–41% reported	SOX17 more specific.
Colorectal adenocarcinoma	0%	Negative	Both useful for exclusion.
Lung adenocarcinoma	0%	Rare positivity	SOX17 more specific.
Urothelial carcinoma	0–1%	Up to 24%	SOX17 more specific.
Pancreatic adenocarcinoma	0%	Rare	SOX17 more specific.
Hepatocellular carcinoma	Rare (0–2%)	Negative	Minimal crossover.
Cholangiocarcinoma	Rare (0–6%)	Rare	Low expression of both.
Mesothelioma	0–17%	Up to ~18%	SOX17 generally more specific but not entirely specific.
Dysgerminoma/Seminoma	75–100%	Usually negative	SOX17 superior.
Yolk sac tumor	73–100%	Usually negative	SOX17 superior.
Angiosarcoma	88–100%	Typically negative	Frequently positive; ERG and CD31 remain the lineage markers.

Abbreviations: PAX8, paired box 8; SOX17, SRY-box transcription factor 17.

## Data Availability

No new data were generated or analyzed for this review.
